# Antitumor Potential and Structure Characterization of Polysaccharides From *Lagotis brevituba* Maxim in the Tibetan Plateau

**DOI:** 10.3389/fnut.2022.921892

**Published:** 2022-07-12

**Authors:** Ruixue Gong, Weiguo Cao, Haijun Huang, Bao Yu, Huan Chen, Wei Tao, Quji Luorong, Juan Luo, Dan Zhang

**Affiliations:** College of Traditional Chinese Medicine, Chongqing Medical University, Chongqing, China

**Keywords:** isolation, purification, structure analysis, antioxidant, antitumor

## Abstract

This study purified two polysaccharides (LBMPs) from *Lagotis brevituba* Maxim in several steps. The chemical structure of LBMP-2 was determined by HPGPC, FT-IR, IC, ^1^H and ^13^C NMR, AFM, SEM, and TEM. The results show that LBMP-2 was mainly composed of GalA, and the Mw of LBMP-2 is 23.799 kDa. In addition, the antioxidant activity, and the antitumor activity *in vitro* and *in vivo* were studied. LBMP-2 has excellent antioxidant and antitumor capacity. The inhibition of tumor cell proliferation *in vitro* may result in the inhibition of aerobic respiration and glycolysis. Tumor growth inhibition *in vivo* may inhibit the expression of AMPK in tumors and enhance spleen function. Compared with conventional chemotherapy drug cyclophosphamide, LBMP-2 is less harmful to the body and safer. Therefore, LBMP-2 provides a potential source of antitumor drugs.

## Introduction

*Lagotis brevituba* Maxim (*L. brevituba*) is a traditional Chinese herb of the Scrophulariaceae family and is mainly distributed over alpine grasslands and gravel slopes at altitudes of 3,000–4,420 m in southwestern Gansu, eastern Qinghai and Tibet (1). At present, there are still relatively few studies on *L. brevituba*, mainly focusing on the chemical components and pharmacological activities.

Due to the plateau situation, plateau plants contain more polysaccharides than ordinary plants. For example, the crude polysaccharides of *Cordyceps sinensis* on the plateau had a total sugar content of 45.6% (2). Recent studies have demonstrated that polysaccharides have strong biological properties. Among them, antioxidant activity (3), antitumor activity and immunomodulatory activity (4) are particularly prominent, and polysaccharides also have fewer toxic and side effects. Therefore, they are widely used in various health products and medicines.

We previously compared the antioxidant activities of *L. brevituba in vivo* and *in vitro* (5), and found that the water extract has strong 1,1-Diphenyl-2-picrylhydrazyl Free Radical (DPPH) free radical scavenging activity. We also found that *L. brevituba* had many polysaccharides. Combined with the relevant modern pharmacological research of *L. brevituba* and polysaccharides, it is speculated that the polysaccharides of *L. brevituba* may have antioxidant activity, regulating immunity and antitumor activity. However, no attention has been given to the characterization or antitumor activity of polysaccharides from *L. brevituba*. Therefore, this study analyzes the structure of the polysaccharides of *L. brevituba*, verifies its antioxidant, immune-regulating and antitumor activities, and explores the relationship between antitumor activity and molecular structure in a more comprehensive manner. Our results provide new structural information on polysaccharides from *L. brevituba* and promote the development and utilization for medicinal use.

## Materials and Methods

### Polysaccharide Isolation and Purification

Following a previous report with some modifications, the whole plant of *L. brevituba* polysaccharides was extracted by boiling water (6).

#### Extraction

*L. brevituba* was shredded and refluxed with 95% ethanol 3 times for 2 h to remove lipids. Then, refluxed with boiling water 3 times for 2 h. A threefold volume of 95% ethanol was mixed with the liquid and left at 4°C overnight. Crude polysaccharides were centrifuged for 10 min at 4,000 rpm and dissolved in 1,000 mL of deionized water.

#### Purification

To separate the water-soluble fraction from the insoluble fraction, a DEAE-cellulose column was used with a gradient of 0–1 mol/L NaCl and a flow rate of 1 mL/min. Eluent (5 mL/tube) was collected automatically. The total carbohydrate content of the elution was determined by the phenol–sulfuric acid method (7). The results showed two large elution peaks, named LBMP-1 and LBMP-2. The samples were dialyzed for 48 h in deionized water with a dialysis bag (molecular weight cutoff 3,500 Da). Under reduced pressure, the samples were concentrated and lyophilized to obtain pure polysaccharides.

### Chemical Analysis

A phenol–sulfuric acid method was used to estimate total carbohydrates (7). The m-hydroxybiphenyl method was used to measure uronic acid (8). Bradford method was used to measure protein content (9). The total polyphenols were determined by using the Follin-Ciocalteu method (10).

### Structural Characteristics of Polysaccharides

#### Molecular Weight

LMBP-2 molecular weight was determined by HPGPC using a Shimadzu HPLC system equipped with a TSKgel G4000PWXL column (7.8 × 300 mm) and a Shimadzu RID-10A refractive index detector (11). The experimental conditions: the detector temperature was 40°C; the column temperature was 35°C; 0.2 mol/L NaCl was used for the elution; the flow rate was 0.3 mL/min, and the injection volume was 10 μL. The standard curve was established using 210, 150, 80, 50, 25, 12, 5, and 1 kDa standard dextran (Aladdin), LabSolutions GPC software was used to calculate the molecular weight of LMBP-2.

#### Monosaccharide Composition

LBMP-2 monosaccharide compositions were determined using high-performance ion chromatography (HPIC) (ICS5000, Thermo Fisher Scientific, United States) and a DionexCarbopac™PA20 column (3 * 150 mm) (12). Trifluoroacetic acid (3 mol/L, 10 mL) hydrolyzed LBMP-2 for 3 h at 120°C in a sealed tube. Then, the hydrolysate was dried with a nitrogen blower at 60°C. Sample dissolved in deionized water at 0.2 mg/mL. After centrifuging the sample solution at 12,000 rpm for 5 min, the supernatant fraction was collected and loaded onto the IC system. Fuc, Ara, Rha, Gal, Xly, Glc, Man, Fru, Rib, GlcA, GalA, GulA, ManA, GalN, GlcN, and GlcNAc were used as standards.

#### FT-IR Analysis

LBMP-2′s FT-IR spectrum was determined with a Spotlight 300 spectrometer (PerkinElmer, United Kingdom).

#### NMR Analysis

A BrukerAscend™600 MHz NMR spectrometer was used to measure the ^13^C and ^1^H NMR spectra of 30 mg of LBMP-2 dissolved in 0.5 mL of D_2_O.

#### Atomic Force Microscope Analysis

BrukerDension Icon atomic force microscopes (AFMs) were used for LBMP-2 AFM imaging. Polysaccharides were dissolved in deionized water and diluted to 3 μg/mL. An AFM was used to image the prepared sample.

#### Scanning Electron Microscope Analysis

SEM imaging was conducted with a scanning electron microscope (SEM) (S-3000N, Hitachi, Japan). LBMP-2 polysaccharides were freeze-dried, fixed with double-sided tape, sputtered with gold, and scanned under a high vacuum.

#### Transmission Electron Microscope Analysis

The sample of LBMP-2 (0.25 mg/mL) was diffused in hot water at 80°C for 10 min, dropped on a carbon-supported copper mesh, dried naturally at room temperature, and observed by a transmission electron microscope (TEM).

### Antioxidant Activity *in vitro*

#### Reducing Ability

The reducing power was determined using the method described previously (13). The positive control was BHT.

#### DPPH Radical Scavenging Activity

The DPPH radical-scavenging activities of LBMP-2 were determined using the method described previously (13). BHT was used as the positive control.

#### ABTS Radical Scavenging Activity

The 2,2′-Azinobis (3-ethylbenzothiazoline -6-sulfonic Acid Ammonium Salt) (ABTS) radical-scavenging activities of LBMP-2 were determined using the method described previously (13). BHT served as a positive control.

### Antitumor Activity *in vitro*

#### Cell Culture

Shanghai Cell Bank of the Chinese Academy of Sciences provided the B16-F10 cell lines.

#### Cell Viability Assay

The antiproliferative activity of the compounds was assessed using the MTT assay (14). 1 × 10^5^ cells/mL were seeded in 96-well microplates. After 12 h of cell culture, the cells were treated with nine concentrations (0, 1.95, 3.9, 7.8, 15.625, 31.25, 62.5, 125, and 250 μg/mL) for 24 h. The 0 μg/mL group was the blank group. Each group had 6 parallel controls.

#### Cellular Metabolic Flux Assay

2.5 × 10^4^ cells/mL were seeded in XFp eight-well cell culture miniplates (Seahorse XF HS Mini, Agilent). LBMP-2 was added to cells at 250 μg/mL for 24 h, then the manufacturer’s protocol was followed during the energy phenotype test, mito stress test and glycolysis stress test (Seahorse XFp Cell Energy Phenotype Test Kit, Seahorse XFp Cell Mito Stress Test Kit, Seahorse XFp Glycolysis Stress Test Kit). The metabolic data of the cells were normalized to the total cellular protein content. Lysates were salvaged and BCA Protein Assay Kit (Beyotime, China) was used to measure total protein.

### Antitumor Activity *in vivo*

#### Animals

A temperature- and humidity-controlled room with a 12-h light/12-h dark cycle was used to maintain mice of male 6-week-old C57BL/6 (20 ± 2 g). All mice were provided with free access to food and water in an SPF-grade environment. The right flank back of C57BL/6 mice was shaved before injection. 4 × 10^5^ cells/mL B16-F10 cells (100 μL) were injected subcutaneously into the right flank of the mice. After 4 days, five groups of mice (*n* = 8) were randomly selected. (1) Model group: normal saline (NS) was intraperitoneally injected into the mice (20 mL/kg); (2) CTX group (Positive group): CTX (cyclophosphamide) was intraperitoneally injected into the mice (20 mg/kg). (3) High dose: LBMP-2 were intraperitoneally injected into the mice (30 mg/kg); (4) Low dose group: LBMP-2 were intraperitoneally injected into the mice (15 mg/kg); (5) Control group: water and food are available for free in an SPF-rated environment. After 11 consecutive days of daily administration, mice were sacrificed with 1% sodium pentobarbital and high-concentration carbon dioxide. A tissue sample was collected for further analysis. Chongqing Medical University’s ethics committee approved all animal husbandry and experimental protocols.

#### Histopathological Examination

As described previously, a histologic examination was performed (15). Paraffin-embedded spleen tissue and tumor tissue were stained with H&E and immunohistochemistry. A primary antibody against AMPK (Servicebio, China) was incubated with tumor tissue sections, and incubation of the spleen tissue sections with primary antibodies against CD3^+^, CD4^+^, CD8^+^ (Servicebio, China). The percent of staining areas was calculated using ImageProPlus.

### Statistical Analysis

All data are expressed as the mean ± standard deviation (S.D.). Statistical analyses were performed by one-way ANOVA using the SPSS statistical package (SPSS. Inc., Chicago, United States). A value of *P* < 0.05 was considered to be statistically significant.

## Results and Discussion

### Extraction, Isolation and Purification of LBMP-2

**FIGURE 1:**
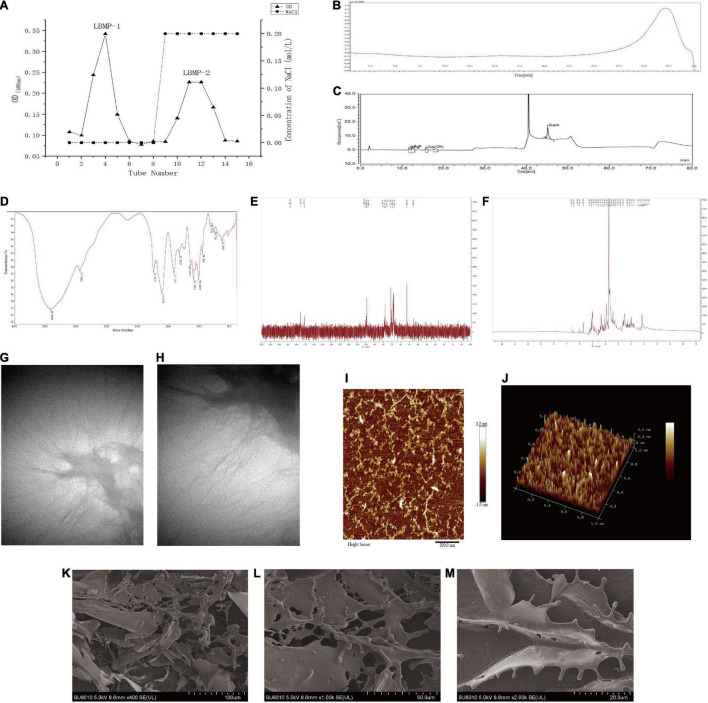
Characterization of LBMP-2. **(A)** Isolation and purification of LBMP-2. **(B)** HPGPC of LBMP-2. **(C)** IC of LBMP-2. **(D)** FT-IR spectra of LBMP-2. **(E,F)** NMR spectra of LBMP-2. **(G–M)** Microstructure analyses of LBMP-2.

The structure of polysaccharides is complex and diverse. Extraction, separation and purification from natural plants are still the main way to obtain polysaccharides. DEAE-cellulose was used to fractionate the LBMP (Figure 1A). The LBMP-1 and LBMP-2 were obtained using a NaCl gradient (0, 0.2 mol/L). The yield of crude polysaccharides was 21.3 ± 1.5%. The purified polysaccharides of LBMP-1 were obtained with a yield of 0.28 ± 0.23% based on the dried whole plant of *L. brevituba*. The purified polysaccharides of LBMP-2 were obtained with a yield of 1.62 ± 0.43% based on the dried whole plant of *L. brevituba*. The content of uronic acid was as high as 67.6 ± 0.32%, and no protein or phenolic acid was detected. The influence of phenolics and proteins on subsequent testing was excluded. Compared with LBMP-2 (1.62 ± 0.43%), the yield of LBMP-1 was low, so no research has been done on LBMP-1.

### Characterization of the LBMP-2

#### Molecular Weight

The molecular weight of polysaccharides was related to their biological activity. HPGPC analysis of LBMP-2 showed a single peak (Figure 1B). LabSolutions GPC software was used to obtain the standard curve: Y = −2.157975e-003X^3^ − 0.1740564X^2^ − 4.843469X + 51.09103; *R*^2^ = 0.9982101. The Mw of LBMP-2 was 23.799 kDa, and the Mn of LBMP-2 was 23.776 kDa. The Mw/Mn ratio was 1, indicating that LBMP-2 was a homogeneous polysaccharide (16).

#### Monosaccharide Composition

In order to determine LBMP-2′s monosaccharide composition, HPIC was chosen instead of GC–MS due to its ability to identify uronic acid with ease (12). As shown in Figure 1C, monosaccharides were composed of Rha, Ara, Gal, Glc, and GalA in molar ratios of 0.156:0.144:0.107:0.114:0.480. Therefore, it can be determined that GalA was the major monosaccharide composition. *In vitro* and *in vivo*, polysaccharides with high uronic acid content, especially high galacturonic acid content, have better antioxidant activity (16, 17).

#### FT-IR Analysis

In FT-IR analysis, LMBP-2 showed absorption peaks characteristic of polysaccharides in the range of 4,000–400 cm^–1^ (Figure 1D). Historically, the broad stretched absorption peak at 3,418 cm^–1^ was considered to be the signature of the O-H bond. At 2,941 cm^–1^, the absorption was caused by the C-H stretching of CH, CH_2_, and CH_3_ groups, which was characteristic of polysaccharides. The peaks at 1,747 and 1,612 cm^–1^ were characteristic absorption peaks of the C = O band for the carboxylate groups. The relatively strong absorption peak at 1,200–1,000 cm^–1^ revealed C-O-C and C-O-H link bonds, which indicated that the pyranose ring was present. The peak at 831 cm^–1^ suggests that the pyranose ring was in the α-configuration. There was no peak at 1,600–1,500 cm^–1^, indicating that protein was not present in LBMP-2. Those results were consistent with the Bradford method (17, 18).

#### NMR Analysis

NMR spectrometer can provide detailed information about polysaccharide structure, including α- or β-anomeric configuration, monosaccharide composition, connection mode, and sugar unit sequence (19). We investigated the structure of LBMP-2 with NMR analysis, in particular its sugar units and linkage patterns. Based on the component, linkage analysis, and literature values, the main chemical shifts of LBMP-2 were found, and they are presented in Table 1. Figures 1E,F show the NMR spectrum of LBMP-2. The signals in the range of δ5.50–4.40 ppm (^1^H NMR) and δ90–110 ppm (^13^C NMR) are assigned to α-D-Glc*p* residues, α-D-Gal*p* residues, α-L-Rha*p* residues, and α-D-Ara*f* residues (19). In the ^13^C NMR spectrum (Figure 1E), the signals in the range of δ170–180 ppm are attributed to the Gal*p*A residue, and the signal in the range of δ52.86 ppm is attributed to the O-CH_3_ group of pectin. In the ^1^H NMR spectrum (Figure 1F), the concentration of chemical shifts at δ3–5 ppm is considered a typical feature of polysaccharides (16), and most α-anomeric protons usually appear at δ5–6 ppm shifts (20). The signal in the range of δ3.82 ppm is attributed to the methyl group in the carboxyl group of GalA, and the signals in the range of δ2.14 and δ2.07 ppm are attributed to the acetyl group at 2-O- and 3-O-GalA. The signal in the range of δ5.18 ppm is attributed to T-ara*f*, and the signal in the range of δ5.08 ppm is attributed to T-α-L-Rha *p* (21, 22). This result also confirms the infrared result.

**TABLE 1:** The ^1^H NMR and ^13^C NMR data of LBMP-2 (in D_2_O solvent).

δ C (ppm)	δ H (ppm)
187.03; 175.01; 170.95 (Gal*p*A)	2.14; 2.07 (2-O- and 3-O-GalA)
52.86 (O-CH_3_ group of pectin)	3.82 (GalA)
100.11	5.08 (T-α-L-Rha*p*)
99.06, 98.94	5.18 (T-ara*f*)
78.79, 77.86	6.58;6.44;6.04
77.44	5.99;5.71
71.27	4.89, 4.60, 4.52, 4,40, 4.31, 4.19, 4.06
68.81	3.69, 3.65, 3.42, 3.27
67.95	2.85, 2.62, 2.29
45.24	1.92, 1.79, 1.48, 1.40, 1.32,1.23, 1.17, 1.10

#### Atomic Force Microscope Analysis

AFM has been widely used in biology and medicine. High-resolution imaging and nanomechanical characterization can be performed to obtain surface topography information, undulation information, roughness information and height information of nanomaterials, polymer materials, biological samples, metal materials, ceramic materials, and thin-film materials (23, 24). AFM was used to analyze the morphology of polysaccharides to gain insight into their chain conformation. Figures 1I,J shows AFM topography images of LBMP-2. Irregular chains could be seen on LBMP-2, its length ranged from 10 to 398 nm, its maximal height was 224 pm, and its maximal width was 15 nm.

#### Scanning Electron Microscope Analysis

The apparent structure of LBMP-2 was studied by SEM (Figures 1K–M). SEM is one of the most effective means of analyzing biopolymer structure, including size, shape, and porosity (25). Figure 1K shows that solid LBMP-2 has a loose and porous sheet structure, and Figures 1L,M clearly shows the highly branched structure of LBMP-2.

#### Transmission Electron Microscope Analysis

The apparent structure of LBMP-2 was studied by TEM (Figures 1G,H). It can be seen in Figure 1G at low magnification (1.4 w times) that polysaccharide chains will form large aggregates through the self-assembly process in water. The random diffusion of polysaccharide chains can be seen under the high magnification lens (2.6 w times) in Figure 1H. This result indicates that the easy aggregation of LBMP-2 in an aqueous solution may be related to the self-assembly process of polysaccharide chains. The random coiling of polysaccharides promotes the interaction between polysaccharides and platelets and can also promote the binding of some specific receptors to cause physiological effects (26).

According to AFM, SEM, and TEM images, LBMP-2 molecules are dendritic multi-branched structures. And it indicated that the spatial structure of LBMP-2 may be a helical structure, and there are many special binding sites. It was reported earlier that either single-helix or multi-helix structure can improve biological immunity and enhance the activity of lymphocytes to achieve the purpose of anti-tumor (27, 28). Therefore, we infer that lbmp-2 had good antitumor activity.

### Antioxidant Activity *in vitro*

**FIGURE 2:**
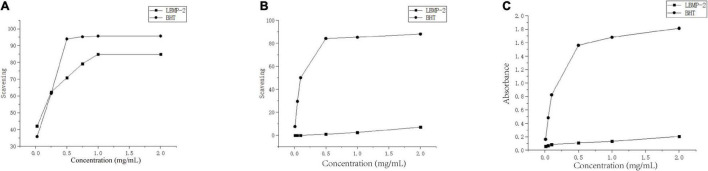
Antioxidant activities of LBMP-2 *in vitro*. **(A)** Scavenging abilities on DPPH radicals. **(B)** Scavenging abilities on ABTS radicals. **(C)** Reducing power.

One of the main causes of aging and disease is oxidative stress. According to the literature, oxidative stress plays a key role in the development and occurrence of cancer (29). Therefore, antioxidants are beneficial for the prevention and treatment of cancer. To determine *in vitro* the antioxidant properties of LBMP-2, DPPH radicals, ABTS radicals and reducing power analysis were conducted, and Figure 2 shows the scavenging ability at concentrations ranging from 0.01 to 2 mg/mL. Figure 2A shows the concentration-dependent scavenging activity of LBMP-2 on DPPH free radicals. The IC_50_ value of LBMP-2 was 0.3 mg/mL, and the IC_50_ value of BHT in the positive control group was 0.26 mg/mL. The DPPH scavenging ability of LBMP-2 showed superior scavenging capacity. Figures 2B,C shows that at a concentration of 2 mg/mL, the scavenging rate of LBMP-2 on ABTS free radicals was only approximately 7%, and the reducing power was much lower than that of BHT.

The reducing power was based on the evaluation method of the single electron transfer mechanism (SET). The DPPH and ABTS methods evaluate the oxidation resistance of the sample based on the hydrogen atom transfer mechanism (HAT) and the single electron transfer mechanism (SET). ABTS prefers the SET evaluation mechanism, while DPPH relies more on the HAT mechanism (30). Based on the different detection principles of the DPPH method, ABTS method, and reduction experiments, LBMP-2 had a superior scavenging capacity *in vitro*. Probably because LBMP-2 contains a lot of uronic acid and lower molecular weight. The uronic acid increased in the number of carboxyl and carbonyl groups and double bonds (31), and the low molecular weights would have a looser structure and more reductive hydroxyl group terminals (32). These special structures increase the possibility of LBMP-2 accepting and eliminating the free radicals.

### Antitumor Activity *in vitro*

**FIGURE 3:**
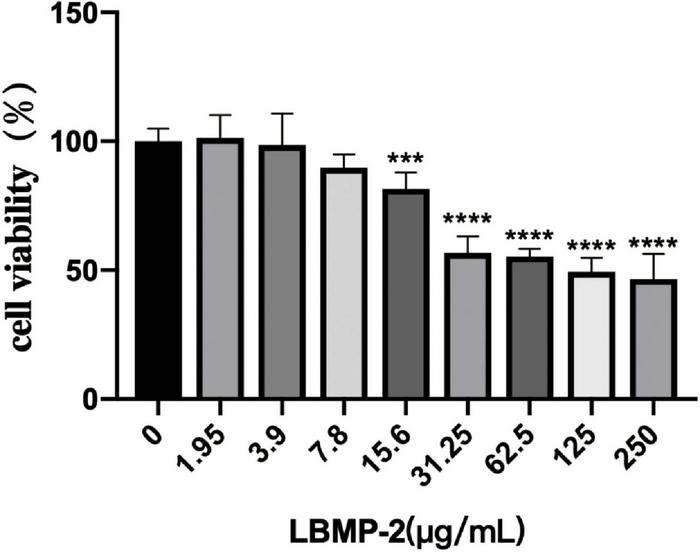
The effect of LBMP-2 on the viability of B16-F10 cells (*n* = 6, ***p < 0.001, ****p < 0.0001).

With increasing LBMP-2 concentration, the viability of B16-F10 cells decreased gradually. After 24 h of intervention, the survival rate of each group is shown in Figure 3. When the LBMP-2 concentrations in the culture medium were 1.95 and 3.9 μg/mL, there was no inhibitory effect on B16-F10 cells. When the concentration was 62.5, 125, 250 μg/mL, the cell survival rate decreased to 55.30, 49.34, and 46.53% (*P* < 0.001). Because of the ability to inhibit cell growth, we chose 250 μg/mL for the follow-up study.

**FIGURE 4:**
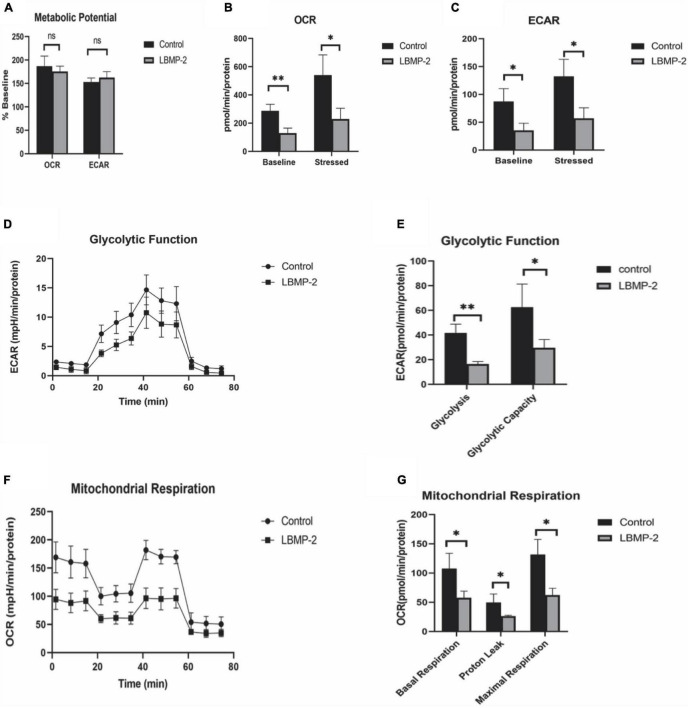
The effect of LBMP-2 on the metabolism of B16-F10 cells. **(A–C)** Energy metabolism phenotype. **(D,E)** Glycolytic metabolism. **(F,G)** Aerobic Respiratory. (*P < 0.05, **P < 0.01, and ns: no significant difference).

With Seahorse XF, two major energy-producing pathways, cellular mitochondrial respiration and glycolysis, can be measured simultaneously. We monitored the OCR (oxygen consumption rate) and ECAR (extracellular acidification rate) in real-time (Figures 4A–C) in response to treatment with LBMP-2 (250 μg/mL) for 24 h in B16-F10 cells. Compared to the control, the treated cells had no significant difference in metabolic potential. However, the OCR and ECAR of the cells were decreased relative to the control. The cell glycolytic ability and mitochondrial function were further tested. LBMP-2 significantly reduced the basal glycolysis and maximal glycolytic capacity compared with the control (Figures 4D,E). LBMP-2 also significantly reduced cell mitochondrial respiration compared with the control (Figures 4F,G), suggesting that LBMP-2 damages the mitochondrial function of B16-F10 cells. Mammalian cells have two key energy metabolism pathways, namely, oxidative phosphorylation (aerobic respiration) and glycolysis. Warburg (33) reported that in malignant tumor cells, glycolysis is the main pathway for their energy supply. LBMP-2 may block the energy supply of cells by simultaneously inhibiting the aerobic respiration and anaerobic respiration of B16-F10 cells, thereby inhibiting cell proliferation.

### Antitumor Activity *in vivo*

**FIGURE 5:**
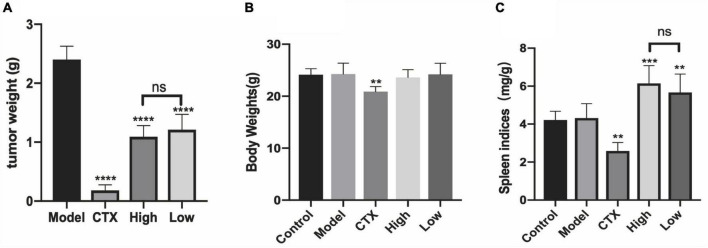
LBMP-2 inhibited tumor growth in melanoma tumor-bearing mice. **(A)** The tumor weight from melanoma tumor-bearing mice; **(B)** the body weight of mice; **(C)** the spleen indices of mice (*N* = 8, **p < 0.01, ***p < 0.001, ****p < 0.0001, and ns: no significant difference).

Regulating the energy metabolism pathway to inhibit tumor growth may be one of the important mechanisms of antitumor drugs. AMPK is the regulating center of energy metabolism balance. It can not only cause an acute metabolic response but also promote metabolic reprogramming and adaptation by regulating specific transcription factors and coactivators (34). At the same time, there are many studies that prove that boosting immunity can inhibit tumor growth (35). To investigate the antitumor activity of LBMP-2 *in vivo*, we subcutaneously implanted a mouse melanoma model. A significant inhibitory effect of LBMP-2 was observed after 11 days of administration. Figure 5 shows that the model group’s average tumor weight was 2.400 ± 0.229 g, while the high dose group was reduced to 1.091 ± 0.1901 g, the low dose group was decreased to 1.460 ± 0.4440 g, and the CTX group was decreased to 0.2588 ± 0.1249 g (Figure 5A). The tumor inhibition rates were 54.53, 39.15, and 89.22%, respectively. In Figure 5B, the mice’s actual body weights were displayed. The CTX group exhibited markedly decreased body weight. The administration group’s spleen index was significantly higher than the model group, while the CTX group’s spleen index was significantly lower (Figure 5C). The results showed that LBMP-2 can effectively increase the spleen index of mice, indicating that LBMP-2 can inhibit the growth of tumors while increasing the immune level of the body, which is safer than CTX.

**FIGURE 6:**
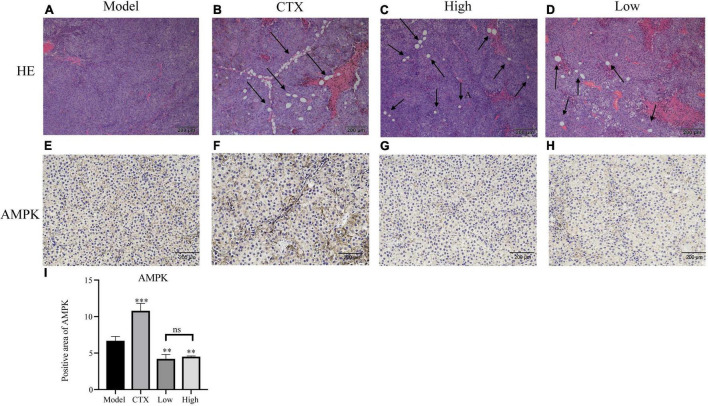
Effects of LBMP-2 on tumors in melanoma tumor-bearing mice. **(A–D)** Representative H&E staining images of tumor sections separated from melanoma tumor-bearing mice. **(E–H)** Representative immunohistochemistry images of tumor sections separated from melanoma tumor-bearing mice. **(I)** Positive area of AMPK (**p < 0.01, ***p < 0.001, and ns: no significant difference).

In order to further assess LBMP-2′s antitumor efficacy, histological H&E staining assays and immunohistochemistry were applied to tumor tissues (Figure 6). A high tumor cell density was seen in the model group, and the nucleus was enlarged and stained deeply (15). Tumors that received CTX or LBMP-2 exhibited massively incomplete structures. According to the data, LBMP-2 inhibits the growth of melanoma tumors in mice. Contrary to the CTX group, there was a significant reduction in AMPK expression in the administration group. AMPK deletion did not accelerate solid tumor growth (36). These results suggest that LBMP-2 inhibits the growth of melanoma by reducing the expression of AMPK. In some cases, AMPK inhibition led to tumor cell death (37). The Seahorse energy metabolism meter found that LBMP-2 can reduce the overall metabolism of B16-F10 cells, which may be achieved by reducing the expression of AMPK.

**FIGURE 7:**
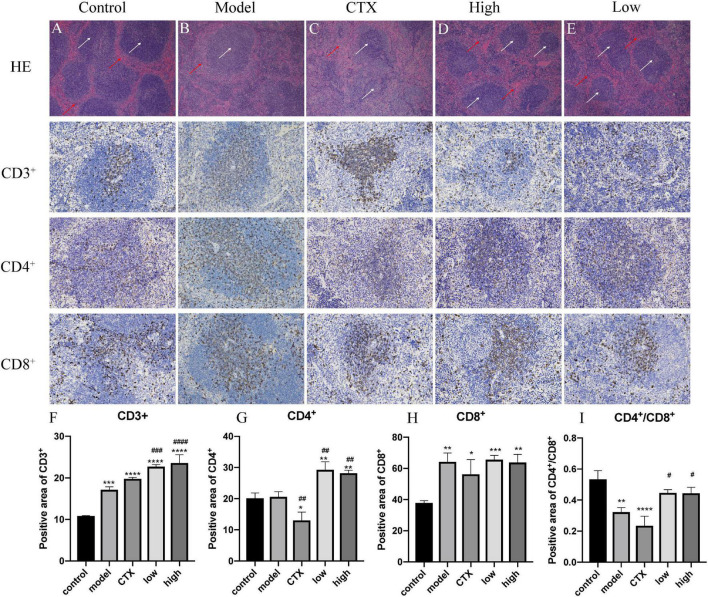
Effects of LBMP-2 on spleen morphology in melanoma tumor-bearing mice. **(A–E)** H&E-staining result, **(F–I)** positive area of immunohistochemistry. *p < 0.05, **p < 0.01, ***p < 0.001, ****p < 0.0001 compared with the control group. ^#^*p* < 0.05, ^##^*p* < 0.01, ^###^*p* < 0.001, ^####^*p* < 0.0001 compared with the model group.

Tumor occurrence and conventional treatment are often accompanied by a decline in immune function (38). The spleen is one of the most important immune organs of the body and the main location of T lymphocytes. The level of the spleen index can evaluate the body’s immune level (39). To determine whether the antitumor effect of LBMP-2 is related to its action on immune organs, histological H&E staining assays and immunohistochemistry were conducted (Figure 7). As shown in Figures 7A–E, the white arrows indicate white pulp, and the red arrows show red pulp. In the spleen tissue, CTX treatment severely damaged the architecture, the cells were arranged loosely. LBMP-2 treatment repaired the cell structure of the white and red pulp, increasing the proportion of white pulp. The immunohistochemistry results showed that compared to the control group, the model group CD4^+^ cells ratio did not change, while the CTX group CD4^+^ cells ratio was significantly lower, while the administration group CD4^+^ cells ratio was significantly higher. The ratio of CD4^+^/CD8^+^ cells decreased in all groups compared to the control group. The CTX group’s CD4^+^/CD8^+^ ratio decreased compared with the model group, whereas that of the administration group increased. No significant difference was found between the doses (Figures 7F–I). T lymphocytes are counted by the CD3^+^ signal. CD4^+^ cells, or Regulatory T cells, play an important role in the immune response. CD8^+^ cells, or suppressor T cells, play the role of negative immunoregulation. Together, they recognize, respond to, and eliminate antigen substances. Immune function can be reflected in their levels. The CD4^+^/CD8^+^ ratio, specifically, was directly related to the immune system’s function (40). This study shows that the increase in CD3^+^ in all experimental groups may be related to the occurrence of tumors that activate the immune system. The spleen index, H&E and immunohistochemical results of the CTX group all showed that cyclophosphamide can destroy the structure of the spleen and reduce the immune function of the body, while these data of the administration group show that LBMP-2 can improve the function of the spleen and enhance immunity. The results suggest that the antitumor effect of LBMP-2 may be related to improving the spleen function of B16-F10 tumor-bearing mice.

## Conclusion

Based on our results, LBMP-2 from *L. brevituba* has antioxidation, inhibits tumor cell proliferation effects *in vitro*, and inhibits tumor growth in tumor-bearing mice. The low molecular weight and high uronic acid content might explain these biological activities. Its antitumor mechanism may be related to inhibiting tumor energy metabolism by down-regulating the expression of AMPK protein while regulating the immune function of the spleen. This research shows that LBMP-2 has potential application value in antitumor drug therapy.

## Data Availability Statement

The original contributions presented in this study are included in the article/supplementary material, further inquiries can be directed to the corresponding author/s.

## Ethics Statement

The animal study was reviewed and approved by the Ethics Committee of Chongqing Medical University.

## Author Contributions

RG: investigation, methodology, and writing—original draft. WC: conceptualization, formal analysis, writing—original draft, funding acquisition, and project administration. HC, WT, QL, JL, HH, and BY: conceptualization, data curation, validation, and methodology. DZ: project administration, supervision, validation, funding acquisition, and writing—review and editing. All authors contributed to the article and approved the submitted version.

## Conflict of Interest

The authors declare that the research was conducted in the absence of any commercial or financial relationships that could be construed as a potential conflict of interest.

## Publisher’s Note

All claims expressed in this article are solely those of the authors and do not necessarily represent those of their affiliated organizations, or those of the publisher, the editors and the reviewers. Any product that may be evaluated in this article, or claim that may be made by its manufacturer, is not guaranteed or endorsed by the publisher.
